# Variability in the incidence of renal replacement therapy over time in Western industrialized countries: A retrospective registry analysis

**DOI:** 10.1371/journal.pone.0235004

**Published:** 2020-06-25

**Authors:** Vicky De Meyer, Daniel Abramowicz, Johan De Meester, Fréderic Collart, Jean-Louis Bosmans, Wilfried Cools, Karl Martin Wissing

**Affiliations:** 1 Department of Nephrology and Hypertension, Vrije Universiteit Brussel (VUB), Universitair Ziekenhuis Brussel, Brussels, Belgium; 2 Department of Nephrology, Universitair Ziekenhuis Antwerpen, Antwerpen, Belgium; 3 Laboratory of Medicine and Pediatrics, Universiteit Antwerpen, Antwerpen, Belgium; 4 Department of Nephrology, AZ Nikolaas, Sint Niklaas, Belgium; 5 Department of Nephrology, CHU Brugmann, Brussels, Belgium; 6 Interfaculty Center Data Processing & Statistics, Vrije Universiteit Brussel (VUB), Brussels, Belgium; University of Liège, BELGIUM

## Abstract

**Introduction:**

A growing number of patients started renal replacement therapy (RRT) in Western industrialized countries between 1980 an early 2000s. Thereafter reports from national and international registries suggest a trend towards stabilization and sometimes a decrease in the incidence rate.

**Aim:**

To investigate the differences in overall and age-specific incidence rates between industrialized countries from 1998 until 2013. Secondly, to investigate changes in incidence rates over time and their association with specific age categories.

**Method:**

We extracted the unadjusted overall incidence of RRT and age-specific incidence rates from renal registry reports in Europe, the United States, Canada, Australia and New Zealand. Time trends in the incidence rate by country and age categories were analyzed by Joinpoint regression analysis.

**Results:**

The incidence rate in 2013 ranged from 89 per million population (pmp) in Finland to 363 pmp in the US. Incidence rates in the lower age categories (20–64 year) were similar between countries and remained stable over time. Higher incidence countries were characterized by higher numbers of patients in both the 65–74 and ≥75 year categories starting RRT.

Joinpoint analysis confirmed that most countries had significant reductions in the incidence rate at the end of the study period. These reductions were explained by lower numbers of older patients starting RRT and were observed also in countries with lower overall incidence rates.

**Conclusion:**

This study confirmed different incidence rates of RRT between industrialized countries worldwide. Countries with the highest overall incidence rates also had the highest incidence rates in the oldest age categories. Since the early 2000’s the number of older patients starting RRT is either stabilizing or even decreasing in most countries. This reduction is universal and is also observed in countries with previously low incidence rates.

## Introduction

Although only a relatively small proportion of the population develops end-stage renal disease (ESRD), renal replacement therapy (RRT) is a chronic resource-intensive process which is responsible for 1–2% of health care spending in high income countries [1]. Therefore, knowledge about factors influencing changes in the incidence of RRT is important for health care planning and the nephrological medical community at large.

Renal registries worldwide collect data on the number of patients starting RRT every year. Reports of these registries show that since 1980 up to the early 2000s the number of patients starting RRT in Western industrialized countries (Australia and New Zealand, Canada, Europe and the US), be it dialysis or preemptive kidney transplantation, continuously increased. In the US the overall unadjusted yearly incidence rate per million population (pmp) increased from 76.3 in 1980 to 326.4 in 2000 [2]. For Canada the incidence rate of RRT rose from 101.5 in 1993 to 155 pmp in the year 2000 [3]. Multiple factors most likely contributed to this growing number of patients with incident RRT, such as an ageing population, the obesity-diabetes epidemic, and the wider access to dialysis therapy across the world [4] [5] [6]. Publications from the ERA-EDTA and USRDS registries suggest that increasing incidence rates have also been to a large extent the consequence of higher numbers of older patients starting RRT [7] [8] [9].

Since the beginning of the 21^st^ century registry reports suggest a trend towards stabilization and even sometimes a decrease in the incidence rate of RRT. A publication from the European registries indicated that the incidence rate of RRT showed a slower increase between 2000 and 2006 as compared to 1997 until 2000 [7]. A subsequent European report then showed a declining incidence rate of RRT between 2008 and 2011 [4]. Less data has been published in peer reviewed journals for non-European populations. US incidence in 2011 was 2.4% lower compared to 2010 [9] but analysis in previous or subsequent years are lacking. Australia and New Zealand reported an incremental incidence rate from 1980 until 2009 [5]. No recent data on the evolution of RRT in Canada are available in the medical literature.

Important differences in the incidence of RRT between countries have been reported. Differences in socio-economic status and health care reimbursement certainly contribute to these differences [10]. An analysis of ERA-EDTA registry data showed that higher numbers of older patients starting RRT were associated with higher overall incidence rates [8]. However, the effect of age on differences in RRT between industrialized countries worldwide has not been investigated.

Comparative data on the evolution of the incidence rate of RRT in industrialized countries worldwide are lacking. Although data from individual countries and Europe are available in registry reports and some scientific publications [4] [5] [6] [7] [9], the format of the reports often makes direct comparison difficult. Based on the available evidence it is therefore unclear whether the reported stabilization in the incidence rates of RRT in the ERA-EDTA registry [4] [7] are a universal phenomenon in Western industrialized countries worldwide. Another question is to what extent the age distribution of patients starting RRT is associated with the evolution of between-country differences in the overall incidence rate of renal replacement therapy over time.

To address these questions, we conducted a retrospective analysis of registry data on the incidence of RRT in a series of Western industrialized countries with similar socio-economic status, universal access to RRT and established patient registries, producing reliable annual counts of incident patients.

The first objective was to describe the differences in overall incidence rate between Western industrialized countries worldwide and to investigate whether these differences were restricted to the older age categories. Our second objective was to evaluate changes in the overall incidence rates over time and their association with the specific age categories.

The study effectively confirmed major between-country differences in overall incidence rates of RRT. These differences were to a large extent explained by variability in the uptake of renal replacement therapies in older patients. Reductions in the overall incidence rates during the most recent period were also the result of lower numbers of older patients starting RRT.

## Methods

### Data collection

Yearly incidence rates of RRT, be it dialysis or preemptive kidney transplantation, were extracted from the registries of the European Renal Association—European Transplant and Dialysis Association (ERA-EDTA www.era-edta.org), the United States Renal Data System (USRDS www.usrds.org*)*, the Canadian Organ Replacement Registry (CORR www.cihi.ca/corr) and the Australia and New Zealand Dialysis And Transplant Registry (ANZDATA www.anzdata.org.au). All these registries publish detailed annual reports on their websites and are publicly available on the websites. Only adult patients with chronic ESRD were taken into consideration.

The mode of data collection by these registries is summarized in S1 Table. For the present study the unadjusted overall incidence rate per million population (pmp) as well as the incidence rate pmp by age categories were collected for the period 1998–2013. Data were not adjusted for age as not all registries publish age adjusted overall incidence rates.

For European countries the data were collected from the ERA-EDTA registry annual reports from the period 1998–2013. The collaborating national and regional renal registries contribute either individual patient data (group A) or aggregated data (group B) [11]. Only countries with 100% of renal centers providing patient-level data since 2000 were included in the present study. Based on these selection criteria, the unadjusted overall incidence rate expressed in patients per million population (pmp) and the country-specific (Austria, Belgium Dutch and French speaking regions, Denmark, Finland, Greece, Norway, Sweden and The Netherlands) incidence rates for different age categories were collected. For the French-speaking region of Belgium, annual data were only available since 2004. Data from the UK, Germany, Spain, Italy and France were not included in the present review because not all dialysis centers in these countries reported patient-level data to the ERA-EDTA.

For the US, all data were extracted from the USRDS annual report of 2015. In this report, ESRD is defined as chronic renal failure requiring RRT to sustain life. A patient is determined as having ESRD when a physician certifies this on the Medical Evidence Report (ME) form, or when there is other evidence of chronic dialysis or a kidney transplant (from other databases) [12].

For Canada data were extracted from the CORR annual report of 2016. An incident patient is defined as a patient with ESRD who began RRT for the first time during the calendar year. Data are collected through a standardized form on an annual basis from all renal units. Because of an underreporting in Quebec from 2011 to 2013, the Quebec data were excluded from the entire dataset [13].

For Australia and New Zealand data were extracted from the ANZDATA annual reports from 1997 until 2015. ANZDATA include all patients receiving RRT where the intention to treat is long-term. The information is collected from all renal units in Australia and New Zealand. Key events (new patients, deaths, transplants) are notified as they occur on standardized forms and are sent to the registry at least monthly. Reports covering a 5-year period are published yearly. This means that between successive reports, incidence rates for individual years can differ slightly. For this reason, the most recently reported unadjusted overall incidence rates pmp for 1998 until 2013, were selected from the reports of 2000 until 2015. The ANZDATA does not report actual incidence rates pmp by age categories, which are shown as figures of the trends over the last 5 years [14]. These data could therefore not be included in the present study.

### Data analysis

Overall and age-specific incidence rates for different countries and age categories are presented in tables. To compare the age-specific incidence rates between different countries, the median incidence rate was calculated between 2004 and 2013 since the data showed that the incidence changed less during this time than in the earlier period.

The evolution of the overall incidence rate and the evolution of the incidence rate by age categories in the time period between 1998 and 2013 was analysed using Joinpoint regression modelling. The Joinpoint analysis identifies the number and location of significant changes in linear trends of incidence rates over time. Within each time interval for each of the age groups the annual percentage change (APC) is obtained with a 95% confidence interval (95% CI) assuming a Poisson distribution as described by Kramer et al [7]. The analysis was performed with the special purpose Joinpoint regression program (version 4.3.1.0; https://surveillance.cancer.gov/joinpoint/).

The evolution of the overall incidence rate in the time period 2004 and 2013 for different age categories incidence rates were also modelled with Poisson regression on the year, age category and country. The relation with the year, was considered linearly but also quadratically and cubic using third order polynomals. Contrary to Joinpoint regression, this model does allow for non-linear changes over time. The fitted evolution was plotted on the observed data.

## Results

### Overall incidence rates of RRT between 1998 and 2013

Overall incidence rates are shown in Table 1. Countries were arbitrarily divided in high (annual incidence rate higher than 150 pmp in 2013) and low incidence countries (annual incidence rate <150 pmp in 2013). Incidence rates reported for 2013 in high incidence countries were in decreasing order (pmp): US 363, Greece 216, Canada 188, then Belgium Dutch speaking 187 and French speaking 183. The low incidence countries reported incidence rates (pmp) of RRT for 2013 being considerably lower as compared to the above-mentioned countries. Austria 142, New-Zealand 125, Denmark 117, Sweden 116, The Netherlands 115, Australia 111, Norway 101 and Finland 89.

**Table 1:** Overall incidence rate (pmp) of renal replacement therapy over time in high incidence countries (above bold line) and low incidence countries (below bold line)^1^^,^^2^.

	1998	1999	2000	2001	2002	2003	2004	2005	2006	2007	2008	2009	2010	2011	2012	2013
US	306	317	323	333	337	340	344	351	362	358	360	368	367	357	359	363
Greece	120	125	157	167	168	180	197	194	198	192	201	205	191	203	210	216
Canada^3^	140	150	155	162	161	162	164	164	166	168	166	169	169	164	156	188
Belgium-Dutch	140	150	149	160	174	175	181	183	192	190	193	207	196	182	188	187
Belgium-French^4^							186	177	187	187	192	199	193	188	188	183
Austria	126	134	132	138	135	140	161	154	160	154	150	150	139	137	140	142
New Zealand	98	98	109	120	119	115	113	111	119	111	116	135	118	110	116	125
Denmark	110	123	132	140	132	132	131	121	119	147	126	133	120	111	124	117
Sweden	127	125	130	127	129	122	123	121	130	128	123	127	121	122	115	116
The Netherlands	94	96	95	101	102	103	106	107	113	117	121	119	117	117	120	115
Australia	86	92	92	98	97	100	97	113	118	113	119	112	106	112	112	111
Norway	90	89	90	95	93	96	101	99	100	113	113	116	104	102	103	101
Finland	90	91	95	91	94	95	97	97	87	94	95	84	84	85	81	89

^1^ pmp: per million population. Data were not adjusted for age as not all registries publish age adjusted overall incidence rates.

^2^ High incidence countries were arbitrarily defined as countries with incidence rates higher than 150 pmp in 2013

^3^ without Quebec

^4^ data only available from 2004

Time trends of the overall incidence rates were quantified using Joinpoint analysis (Table 2). The evolution of incidence rates, estimated by annual percentage change (APC) varied considerably among countries. In ten out of the thirteen countries incidence rates of RRT first increased during a variable number of years after 1998. This initial increase was observed in all high incidence countries (Table 2). During the first decade of the years 2000, incidence rates stabilized in all high incidence countries except Greece (Table 2). Remarkably, three out of seven low incidence countries did not experience the early increase in incidence rates that were observed in the high incidence countries. Although starting from lower incidence rates, the number of patients starting RRT further decreased from 1998 in Sweden and Finland and remained stable in Denmark. Interestingly, Austria and Norway who had positive slopes of incidence rates during the first years after 1998 had significant reductions in the annual percentage changes respectively after 2006 and 2009. The reduction in RRT incidence rates observed in Norway (APC between 2009–2013–3.2% (95%CI -5.9 to -0.3)) was the largest observed in all investigated countries.

**Table 2:** Annual percentage change and 95% confidence interval of the overall unadjusted incidence rate of renal replacement therapy by country as analysed by Joinpoint regression.

Country	Joinpoint 1		Joinpoint 2		Jointpoint 3	
	Periode	APC % (95% CI)	Periode	APC % (95% CI)	Periode	APC % (95% CI)
High						
US	1998–2006	1.9 (1.5 to 1.3)	2007–2013	0.1 (-0.4 to 0.5)^$^		
Greece	1998–2003	9.0 (5.5 to 12.6)	2003–2013	1.1 (0.1 to 2.0)^$^		
Canada *	1998–2001	4.7 (2.9 to 6.6)	2001–2011	0.5 (0.2 to 0.8)^$^	2011–2013	2.7 (-0.5 to 6.1)
Belgium-Dutch	1998–2006	4.1 (2.8 to 5.4)	2006–2013	-0.5 (-1.9 to 0.9)^$^		
Belgium-French	2004–2009	1.9 (1.1 to 2.8)	2009–2013	-1.7 (-4.8 to 1.6)^$^		
Low						
Austria	1998–2006	2.8 (1.6 to 4.1)	2006–2013	-2.1 (-3.6 to -0.6)^$^		
New-Zealand	1998–2013	1.0 (0.3 to 1.8)				
Denmark	1998–2013	-0.3 (-1.3 to 0.6)				
Sweden	1998–2013	-0.5 (-0.8 to -0.2)				
The Netherlands	1998–2008	2.6 (2.1 to 3.0)	2008–2013	-0.3 (-1.5 to 0.8)^$^		
Australia	1998–2006	3.6 (2.2 to 5.0)	2006–2013	-0.3 (-1.8 to 1.2)^$^		
Norway	1998–2009	2.3 (1.7 to 3.0)	2009–2013	-3.2 (-5.9 to -0.3)^$^		
Finland	1998–2013	-0.7 (-1.2 to -0.1)				

* without Quebec

^$^ significant reduction in incidence rate as compared to previous period in Joinpoint analysis (slope below lower 95% CI of previous estimate)

Joinpoint analysis identifies points in time where a significant change in the linear slope of a trend occurs. Between these time points, the annual percentage change (APC) is considered constant and provided with 95% confidence intervals (95% CI). The number and location of the Joinpoints are determined by the data

### Age-stratified incidence rates of RRT per country

Median incidence rates for the period from 2004 to 2013 were stratified by country and age category (Table 3). It was notable that, with the exception of the US, incidence rates in the lower age categories (20–64 year-old) were similar between countries. Higher incidence countries were characterized by larger numbers of incident patients in both the 65–74 year and ≥75 year age categories. In European countries with high overall incidences of RRT, 17.0 to 21.6 times more patients in the ≥75 started RRT than in the 20–44 year age category whereas this ratio was only 5.6 to 13.0 in the low incidence countries (Table 3). High incidence countries also had higher numbers of patients in the ≥75 as compared to the 65–74 year category, whereas incidence rates in these two categories were similar in low incidence countries (Table 3). Finland, the country with the lowest overall incidence rate, was also the only country with less patients starting RRT in the ≥75 as compared to the 65–74 year category between 2004 and 2014 (Table 3).

**Table 3:** Median incidence rates (pmp*) of RRT between 2004 and 2013 by age category and country.

Country	20–44 y	45–64 y	65–74 y	≥ 75 y	Overall	Rate ratio ≥75 y	Rate ratio ≥75 y
	Median (Min Max)	Median (Min Max)	Median (Min Max)	Median (Min Max)	Median (Min Max)	vs 20–44 y	vs 65–74 y
High							
US	125.8 (120.3–130.1)	531.9 (516.3–541.2)	1241.5 (1142.7–1297.3)	1530.7 (1396.3–1555.3)	359.1 (343.5–368.2)	12.2	1.2
Greece	40.8 (37.3–44.6)	189.6 (180.2–205.9)	573.1 (501.4–626.6)	880.5 (822.7–908.5)	199.4 (190.7–215.8)	21.6	1.5
Canada^$^	58.1 (53.4–60.2)	222.2 (211.4–235.6)	596.8 (572.0–653.4)	783.1 (727.7–810.6)	176.5 (171.8–187.9)	13.4	1.3
Belgium (Dutch)	42.3 (38.0–45.5)	165.8 (147.6–177.9)	535.0 (464.5–606.2)	903.2 (822.0–984.2)	189.0 (181.4–207.2)	21.4	1.7
Belgium (French)	49.5 (33.7–57.5)	210.0 (190.4–234.7)	594.3 (491.4–649.3)	841.9 (813.9–894.0)	187.4 (176.8–199.1)	17.0	1.4
Low							
Austria	38.8 (36.9–44.5)	188.6 (152.1–247.5)	473.1 (391.4–517.6)	506.7 (452.5–550.9)	150.0 (137.4–160.8)	13.0	1.1
Denmark	46.3 (39.9–54.4)	151.0 (124.2–179.2)	360.6 (314.6–435.1)	456.9 (386.8–534.6)	122.8 (111.1–147.2)	9.8	1.3
Sweden	44.5 (32.5–48.2)	146.9 (130.5–156.2)	357.0 (306.2–396.3)	409.3 (357.1–441.7)	122.6 (114.5–129.9)	9.2	1.1
The Netherlands	42.3 (37.7–44.5)	135.6 (122.9–146.6)	375.7 (350.4–392.4)	458.4 (365.8–529.9)	117.0 (105.7–120.7)	10.8	1.2
Norway	44.3 (35.8–52.8)	130.6 (107.7–155.9)	336.7 (278.5–380.8)	392.5 (382.2–429.9)	102.2 (99.5–116.4)	8.9	1.2
Finland	39.5 (29.2–50.8)	120.0 (105.9–134.1)	244.0 (199.8–284.3)	222.1 (172.5–266.4)	87.9 (80.9–97.4)	5.6	0.9

*pmp: per million population

^$^ Without Quebec

**Fig 1:**
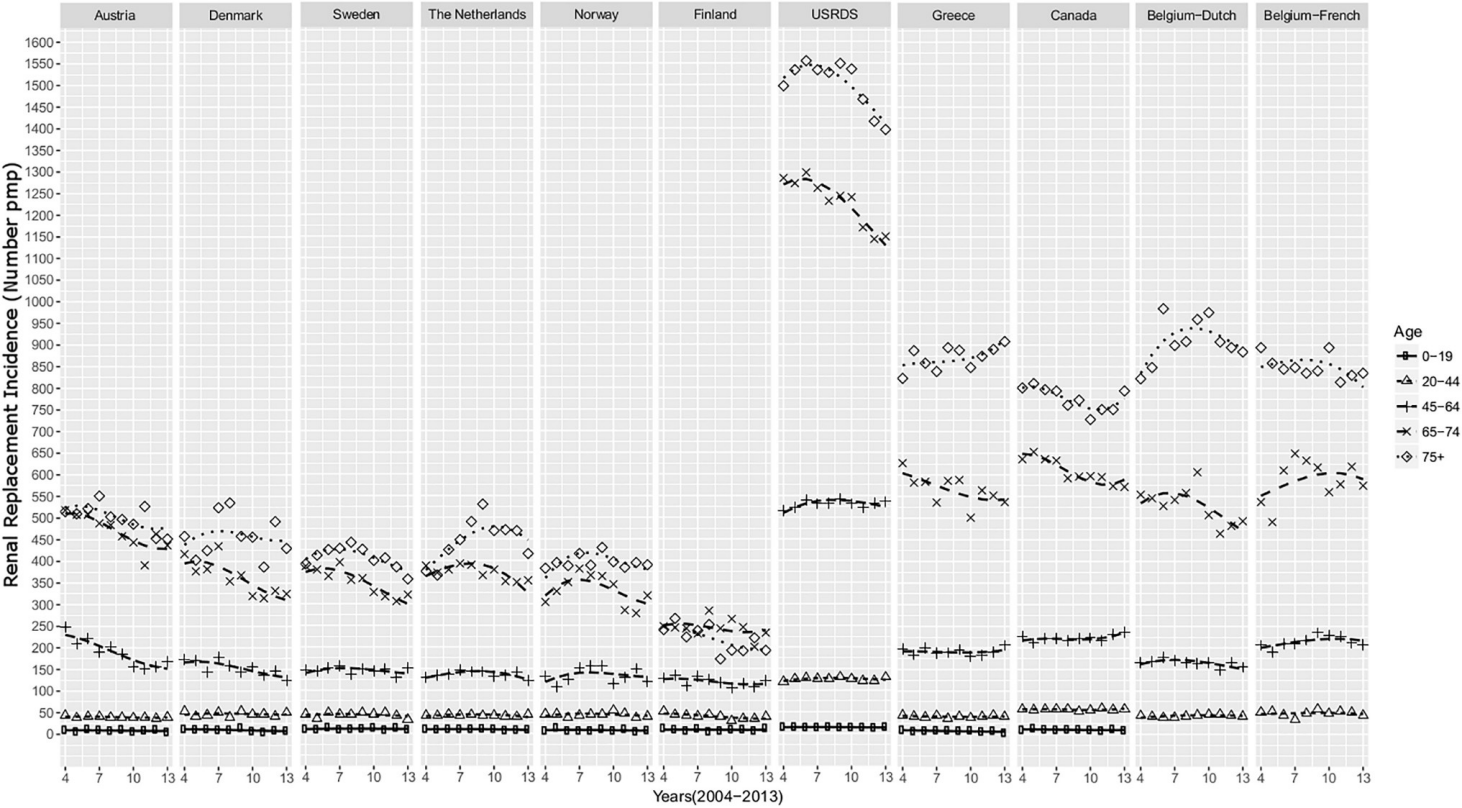
Overall incidence rate pmp*of RRT by country and age category from 2004 until 2013. The symbols represent the actual incidence rate while for each combination of country and age category, a line is included to show an estimated change over the years. For the estimation, the incidences are modelled with polynomial regression on the year, the age category and the country. *pmp: per million population.

Evolution of time trends of incidence rates in the older age categories, fitted by polynomial regression modelling, are presented in Fig 1 (Fig 1). These data show, with the notable exception of the US, that incidence rates for patients under the age of 65 were similar between countries and relatively stable over time (Fig 1). Incidence rates the older age categories peaked around the middle of the first decade of the new millennium in the majority of countries (Fig 1). By 2013, incidence rates decreased in all countries except in Greece for the ≥75 year category (table in S2 Table). Median reductions in fitted incidence rates were -18.6% (95%CI -29.0 to -2.4%) for the 65–74 year category and -7.9% (95%CI -27.3 to 0%) in the ≥75 year category. Incidence rates decreased during the same periods and in similar proportions in both high and low incidence countries (table in S2 Table).

This was also confirmed by age-stratified linear regression using Joinpoint modelling which showed almost no significant change in incidence rates over time in the younger age categories in all countries (Table 4). In the countries that showed a significant increase or decrease the APC was usually low.

**Table 4:** Annual percentage change (APC) and 95% confidence interval (95% CI) of the incidence rate by country and age category as analysed by Joinpoint regression.

Country	Periode	APC % (95% CI)	Periode	APC % (95% CI)	Periode	APC % (95% CI)
≥75 y						
High						
US	1998–2002	5.1 (3.6 to 6.6)^1^	2002–2009	0.7 (0.0 to 1.5)	2009–2013	-2.9 (-4.2 to -1.6)^2^
Greece	1998–2001	34.1 (25.1 to 43.6)^1^	2001–2004	11.1 (1.0 to 22.2)^1^	2004–2013	0.6 (-0.2 to 1.4)
Canada^$^	1998–2001	13.2 (7.9 to 18.8)^1^	2001–2013	-0.6 (-1.1 to -0.1)^2^		
Belgium-Dutch	1998–2006	8.2 (4.3 to 12.1)^1^	2006–2013	-1.1 (-4.8 to 2.7)		
Belgium-French†			2004–2013	-0.5 (-1.2 to 0.2)		
Low						
Austria	1998–2005	9.2 (6.7 to 11.7)^1^	2005–2013	-1.7 (-3.3 to -0.1)^2^		
Denmark‡			2001–2013	0.8 (-1.0 to 2.6)		
Sweden‡			2001–2008	1.3 (-0.1 to 2.6)	2008–2013	-3.3 (-5.5 to -1.0)^2^
The Netherlands	1998–2009	7.0 (5.7 to 8.4)^1^	2009–2013	-4.7 (-9.6 to 0.5)		
Norway	1998–2005	9.2 (6.5 to 11.9)^1^	2005–2013	-0.1 (-1.8 to 1.7)		
Finland‡			2001–2013	-0.6 (-3.3 to 2.2)		
65–74 y						
High						
US	1998–2007	1.3 (0.5 to 2.1)^1^	2007–2013	-1.9 (-3.4 to -0.5)^2^		
Greece	1998–2003	8.4 (4.0 to 12.9)^1^	2003–2013	-1.0 (-2.3 to 0.2)		
Canada^$^	1998–2002	3.2 (1.5 to 5.0)^1^	2002–2010	-1.6 (-2.3 to -0.9)^2^	2010–2012	-7.7 (-12.9 to -2.2)^2^
Belgium-Dutch	1998–2013	0.2 (-0.9 to 1.4)				
Belgium-French†			2004–2013	0.8 (-1.4 to 3.0)		
Low						
Austria	1998–2013	-0.3 (-1.2 to 0.6)				
Denmark‡			2001–2013	-4.0 (-5.0 to -2.9)^2^		
Sweden ‡			2001–2013	-1.6 (-2.6 to -0.7)^2^		
The Netherlands	1998–2007	1.3 (0.5 to 2.1)^1^	2007–2013	-1.9 (-3.4 to -0.5)^2^		
Norway	1998–2013	0.1 (-1.1 to 1.4)				
Finland‡			2001–2013	-1.4 (-2.9 to 0.1)		
45–64 y						
High						
US	1998–2013	0.4 (0.3 to 0.6)^1^				
Greece	1998–2013	0.5 (-0.2 to 1.3)				
Canada^$^	1998–2013	-0.2 (-2.5 to 2.1)				
Belgium-Dutch	1998–2013	-0.5 (-1.2 to 0.3)				
Belgium-French	2004–2010	2.9 (0.4 to 5.5)^1^	2010–2013	-3.9 (-10.6 to 3.3)		
Low						
Austria	1998–2004	2.7 (-0.9 to 6.4)	2004–2013	-4.4 (-6.3 to -2.4)^2^		
Denmark‡	2001–2013	-2.6 (-3.8 to -1.5)^2^				
Sweden ‡	2001–2013	-0.7 (-1.6 to 0.2)				
The Netherlands	1998–2013	-0.3 (-0.9 to 0.3)				
Norway	1998–2013	0.5 (-0.7 to 1.8)				
Finland‡	2001–2013	-1.7 (-3.1 to -0.4)^2^				
25–44 y						
High						
US	1998–2013	0.3 (0.0 to 0.6)				
Greece	1998–2013	-0.5 (-1.1 to 0.1)				
Canada^$^	1998–2013	-0.3 (-0.9 to 0.3)				
Belgium-Dutch	1998–2013	1.2 (0.4 to 2.0)^1^				
Belgium-French	2004–2013	0.1 (-3.4 to 3.8)				
Low						
Austria	1998–2013	-1.6 (-2.2 to -1.0)^2^				
Denmark‡	2001–2013	-0.1 (-1.8 to 1.7)				
Sweden ‡	2001–2013	-0.8 (-2.8 to 1.1)				
The Netherlands	1998–2013	-0.3 (-0.9 to 0.4)				
Norway	1998–2013	0.0 (-1.5 to 1.4)				
Finland‡	2001–2013	-2.4 (-4.0 to -0.7)^2^				

^†^ data only available from 2004

^‡^ data only available from 2001

^$^ without Quebec

^1^ significant increase in incidence rate

^2^ significant decrease in incidence rate

Joinpoint analysis identifies points in time where a significant change in the linear slope of a trend occurs. Between these timepoints, the annual percentage change (APC) is considered constant and provided with 95% confidence intervals (95% CI). The number and location of the Joinpoints are determined by the data.

Evaluation of time trends in the older age categories was hampered by the fact that age-specific incidence rates were available for some countries only from 2001 (Table 4). All countries with data available from 1998 reported major increases in the incidence rate of the ≥75 year category during the first years of the observation period. Annual percentage changes increased 5.1% (3.6 to 6.6) in the US and 34.1% (25.1 to 43.6) in Greece. Subsequently, incidence rates in the oldest age category stabilized in all countries and even decreased significantly in the US, Canada, Austria and Sweden (Table 4). Increases in incidence rates for the 65–74 year category were less pronounced but either stable or decreasing incidence rates over time were observed in all countries (Table 4).

## Discussion

Our data confirmed the very large differences in patients starting RRT among Western industrialized countries, with an approximately four-fold higher global incidence in the US as compared to Finland, the countries with respectively the highest and lowest incidence rates. These startling differences in incidence rates have been reported previously in Europe [4] [7] [8] and worldwide [10]. Our study confirms that very large differences persist among countries selected for a similar level of socio-economic development, comparable overall life expectancy and universal access to RRT.

Incidence rates were similar for patients younger than 65 years in all countries except the US. On the contrary, high incidence countries, defined by an overall incidence rate above 150 pmp in 2013, were characterized by higher numbers of patients in the 65–74 year and ≥75 year age categories starting RRT. Interestingly, whereas low incidence countries had similar incidence rates in these two age categories, in high incidence countries considerably more patients of ≥75 years start RRT as compared to the 65–74 year category. Our observation confirms, on a global level, data report of the European ERA-EDTA registry which had also documented that the median age at start of RRT is strongly correlated with the overall incidence of RRT [8].

The reasons for the major differences in the number of older patients entering RRT are unclear. It is possible that variations among countries in prevalence and control of conditions such as diabetes and hypertension might account for some of the observed differences [15]. Furthermore differences in chronic kidney disease management resulting in higher numbers of patients reaching ESRD at older age, might contribute [15]. However, it appears unlikely that such putative differences in disease prevalence and in the quality of medical care could fully explain the more than two-fold higher incidence of RRT in countries with otherwise similar population demographics and health systems. With the exception of the US, the incidence rates in the younger age categories were comparable between the countries under investigation, although we observed major between-country differences in patients older than 65 years, thus suggesting that age at start of dialysis is a major determinant of the large differences in overall incidence rates among these countries.

In addition, countries might differ in the proportion of older patients with CKD grade 5 who effectively start RRT. A study linking ANZDATA registry data of end stage renal disease to the registration of end stage renal disease in the Australian death registries, showed that patients younger than 65 years with advanced renal failure nearly always received RRT, whereas the proportion of patients effectively receiving RRT decreased sharply in older patients, with only 4% of new cases of 85 years or older being treated [16]. A similar analysis in Canada confirmed that older patient with ESRD are less likely to start with RRT compared with younger patients [17]. The higher median age in high incidence countries and the strong correlation between median age at start of RRT and the overall incidence of RRT in countries contributing data to the ERA-EDTA registry, are strong arguments to conclude that different practice patterns concerning RRT in the elderly are to a large part responsible for between-country differences in the overall number of patients on RRT [8]. It has been suggested that in patients aged ≥75 years with multiple extra-renal comorbidities, the survival advantage of RRT over conservative care is likely to be small [18]. A European survey study in 2013 showed that 8% of nephrologist in low incident countries offer RRT to patients even when they anticipate that live expectancy and quality of live will not improve vs 20% of nephrologists in high incidence countries [19]. Different uptake of conservative renal care could therefore be a possible explanation for the between-country differences in RRT in the elderly we observed in our study.

Differences in residual renal function at start of RRT could also have a substantial impact on RRT incidence [20]. In older subjects loss of renal function occurs slower and eGFR often decreases by less than 2 ml/min/year [21] [22]. Therefore starting RRT at lower residual renal function can substantially increases RRT-free survival and consequently the risk of death before starting RRT [9, 17, 22]. RRT start with higher residual function has been shown to be closely correlated with higher incidence rates of RRT at population level [20]. Between 2005 and 2015 the frequency of early start dialysis, defined as predialysis estimated glomerular filtration rate > 10 ml/min/1.73m^2^, was 38.8% in the US, 37.2% in Canada and 21.7% in Australia [23] correlating with the higher overall incidence in this study in the US and Canada as compared to Australia. Further investigation of between-country differences in residual renal function at start of RRT and their role in the observed differences in the number of patients receiving RRT is clearly required.

A second objective of our study was to investigate whether changes in the incidence of RRT over time varied among countries and to investigate whether this variability was linked to patient age. Joinpoint analysis clearly showed that the first part of the follow up period extending from the 1990s to the first decade of the new millennium, has been characterized by a steep increase in the number of patients starting RRT in the majority of countries, in particular all high incidence countries. However, this increase did not occur in Denmark, Sweden and Finland. Finland is the only country with decreasing incidence rates of RRT during the whole period (1998 to 2013) and in addition the country with the lowest overall incidence rate.

Joinpoint analysis also showed that subsequently, at variable moments during the first decade after the turn of the century, all countries, except New Zealand, Denmark, The Netherlands and Finland, have seen significant reductions in the growth rates of patients receiving RRT. These exceptions were all low incidence countries having either already negative slopes or low positive annual changes (New Zealand) early during the observation period.

Analysis of the evolution of incidence rates by Joinpoint analysis and non-linear modelling after stratification for age, confirmed that incidence rates in patients under 65 years of age remained stable over time and that changing patterns during the 15-year period of registry data were clustered in the two oldest age categories. Data from the ERA-EDTA registry have linked the increase in overall incidence rates between 1997 and 2006 to higher numbers of patients older than 74 years starting RRT, whereas the numbers of younger patients remained relatively stable [7]. Similarly, in the US the huge increase in the overall number of patients on RRT between 1996 and 2005 was to a large extend caused by the 80% increase in incident RRT patients in the ≥75 age category during this period [9].

To our best knowledge our study is the first reporting a stabilization and even reduction in the incidence rate of RRT in Western industrialized countries worldwide. As for the previously observed increase, the reduction in the incidence rate of RRT was again clustered in the older age categories. Countries with strong growth rates in the number of incident ≥75 patients starting RRT at the start of the observation period (the US, Greece, Canada, Belgium Dutch speaking, Austria, The Netherlands and Norway) all later experienced marked reductions in the slopes of annual percentage changes. A surprising observation of our study was that the relative reduction in incidence rates was observed both in high and low incidence countries. Proportional reductions in the older age categories even tended to be more important in low incidence countries. The observed changes therefore do not only represent a correction after a period of excessive growth, but reflect a broad trend which appears independent from background risk.

The reasons for the decreasing numbers of older patients starting RRT in the more recent period are unknown. Between 1996 and 2009 the marked increase in patients aged ≥75 years starting RRT in the US has been accompanied by a parallel increase in the proportion of older patients starting RRT with an eGFR > 10ml/min/1.73m^2^ from 25% to more than 60% [9]. The prospective and randomized IDEAL study was published in 2010 and has provided solid evidence that starting RRT based on clinical symptoms rather than estimated creatinine clearance can significantly delay the start of dialysis without increase in mortality or adverse events [24]. Decreasing incidence rates during the last years in older recipients might therefore reflect that RRT is started at lower levels of residual renal function [20].

Several reports during the early 2000s suggested that in patients aged ≥75 years with multiple extrarenal comorbidities are unlikely to benefit from RRT in terms of the survival time outside the hospital setting and quality of life [25] [26] [27]. Patient and families are increasingly implicated in planning of care, and information about the possibility of conservative renal care by health care providers [28] [29]. It is likely that these factors will influence the uptake of conservative care over time.

Finally, several studies have reported that the recent incidence changes of RRT are due to a decrease in patients diagnosed with diabetic nephropathy as the primary cause of ESRD [5] [30]. This means that changes in the prevalence of diabetes, improved interventions that slow the rate of progression of diabetic nephropathy and changes in competing risks of mortality may influence RRT incidence [31]. An ERA-EDTA study of 2009 showed that the slowing growth in incidence rates of RRT due to diabetes and renal vascular disease was caused by changes in the age groups 65–74 and 75–84 year [7].

A strength of our study is that we use registry data based on unselected large patient populations originating from several industrialized countries with universal access to RRT from all over the world. To analyze whether fluctuating incidence rates represent random variability or reflect real changes in the underlying epidemiology of RRT requires regression analysis of the slopes of modelled incidence rates. A drawback of Joinpoint regression is that the method postulates linear evolutions of incidence rates over time with changes of the slope at specific time points [7]. We complemented this analysis with non-linear modelling of incidence rates using third degree polynomials. The fact that both methods confirmed slowing in the increase or even reductions in the incidence rate of RRT starting during the first decade of the new millennium in the majority of countries increases the internal validity of these conclusions.

A limitation of our study is the restriction to data publicly available in the annual reports of different registries. The combination of data originating from different registries is also the reason that crude and not adjusted incidence rates were used. We tried to minimize confounding by focus our analysis on age categories. The descriptive nature of our analysis suggests that the age distribution at start of RRT is a major determinant of the marked differences in incidence rates between countries and of their evolution over time, but does not allow to confirm a causal link and was unable to quantify additional factors conditioning these outcomes. The present study included publicly accessible registry data from a worldwide selection of Western countries with high socio-economic development and universal access to RRT. A limitation of our study is that many countries fulfilling these conditions have not been included into the present analysis, which does therefore not provide a fully global perspective. This is in particular true for several Asian countries with universal access to RRT, such as Japan, the Republic of Korea, Taiwan, Thailand and Malaysia. These countries also differ in terms of incidence rates of RRT and in the age distribution of incident patients [12]. Whether higher overall incidence rates are also linked to a higher proportion of older patients starting RRT in these countries certainly merits investigation. A further shortcoming of the study is the end of the observation period in 2013. Whether between country-differences in the incidence rate of RRT and in the number of older starting RRT have changed since has to be confirmed by additional registry analysis.

To further understand and analyze the differences in the incidence rate of RRT between countries and over time it is necessary to collect data on the residual renal function at the start of RRT. In addition this should be complemented by data on the prevalence of chronic kidney disease KDIGO stage 4 and 5 without dialysis therapy in the different countries. Ideally these data should than be combined with a qualitative research on factors conditioning the uptake of conservative therapy by both patient and health care professionals.

## Conclusion

The present study confirms striking differences between Western industrialized countries worldwide in the incidence rates of RRT in the age category 65–74 year and ≥75 year. Countries with the highest overall numbers of patients starting RRT also have the highest incidence rates in the oldest age categories.

Since the start of the new millennium, the number of patients starting RRT is stabilizing, and even decreasing in some countries. These changes over time are caused by decreasing incidence rates in older patient categories.

The reasons conditioning these large differences between countries and changes over time are unclear. Given the implications in terms of survival and quality of life for patients as well as cost and resources for health care systems, there is a major need to investigate the interplay of factors conditioning the start of RRT in elderly patients.

## Supporting information

S1 Table Characteristics of data collection in different registries. (DOCX)

S2 Table Maximum fitted average of incident dialysis versus fitted average at 2013 by age category and country. The evolution of the overall incidence rate in the time period 2004 and 2013 for different age categories incidence rates were modelled with Poisson regression on the year, age category and country. The relation with the year was considered both linearly and using third order polynomals.
(DOCX)
