# Supplementary material for: Variability in the incidence of renal replacement therapy over time in Western industrialized countries: A retrospective registry analysis
Source: PLoS One. 2020 Jun 25;15(6):e0235004. doi: 10.1371/journal.pone.0235004 (PMC7316284; doi:10.1371/journal.pone.0235004)
Supplement: S1 Table — (DOCX) [file pone.0235004.s001.docx]

**S1 Table: Characteristics of data collection in different registries**

|  | ERA-EDTA | USRDS | CORR | ANZDATA |
| --- | --- | --- | --- | --- |
| Definition incident patients | - Patient starting RRT in that year (some countries dialysis alone) | - Patient diagnoses with ESRD* in that year | - Patient starting RRT in that year - AKF = excluded | - Patient starting RRT in that year - AKF = excluded |
| Data collection incident patients | - Annual data from some national and regional registries in Europe - Divided in 2 groups - A: individual patient data - B: aggregated data - Not all registries submit complete data sets of incident patients every year - Volontary | - US - Physician certifies ESRD on the ME form or other evidence of chronic dialysis or a kidney transplant - AKF are excluded when ME forms are not submitted - 100% of renal units (since 1995) - Mandatory | - Canada - Annual data sending - Web based spreadsheet or paper - 100% of renal units but missing data from Quebec 2011 and 2012 - Voluntary | - Australia and New Zealand - Physician decides if there is the intention of long term treatment - New ESRD patients are registered as they occur - Web based or on paper (fax or post) - 100% of renal units - Voluntary |
| Report   - Incident data - Primary renal disease - Data < 20y | Annual  Day 1 and Day 91 RRT (some HD only)  ERA-EDTA (1994-1995)   - Diabetes - Hypertension - Glomerulonephritis - Polycystic - Renal vascular disease - Pyelonephritis - Other - Unknown   Not for all registries | Annual  Day 1 RRT  8 categories ICD9-CM   - Diabetes - Hypertension - Glomerulonephritis - Cystic kidney disease - Other urological - Other - Unknown - Missing   Yes | Annual  Day 1 RRT   - Diabetes - Glomerulonephritis - Polycystic - Renal vascular disease - Pyelonephritis - Drug induce - Other - Unknown   Yes | Annual  Day 1 RRT  Modified EDTA coding system   - Diabetes - Hypertension - Glomerulonephritis - Polycystic kidney disease - Reflux - Analgesic nephropathy - Miscellaneous - Uncertain   Yes |
| Years | 1998-2012 | 1980-2012 | 1993-2012 | 1992-2012 |

ERA-EDTA: European Renal Association – European Transplant and Dialysis Association

List of ERA-EDTA member registries: <https://www.era-edta-reg.org/index.jsp?p=10>

USRDS: United States Renal Data System

CORR: Canadian Organ Replacement Registry

ANZDATA: Australia and New Zealand Dialysis and Transplant Registry

AKF: acute kidney failure

ME form: the medical evidence report form

* ESRD is defined as chronic renal failure requiring RRT to sustain life

Only data from ERA-EDTA country/regional registries with patient level data and full reporting during the study period were included into the present study.
