# Supplementary material for: Variability in the incidence of renal replacement therapy over time in Western industrialized countries: A retrospective registry analysis
Source: PLoS One. 2020 Jun 25;15(6):e0235004. doi: 10.1371/journal.pone.0235004 (PMC7316284; doi:10.1371/journal.pone.0235004)
Supplement: S2 Table — The evolution of the overall incidence rate in the time period 2004 and 2013 for different age categories incidence rates were modelled with Poisson regression on the year, age category and country. The relation with the year was considered both linearly and using third order polynomals. (DOCX) [file pone.0235004.s002.docx]

**S2 Table: Maximum Fitted Average of incident dialysis Versus fitted average at 2013 by Age Category and Country**

The evolution of the overall incidence rate in the time period 2004 and 2013 for different age categories incidence rates were modelled with Poisson regression on the year, age category and country. The relation with the year was considered both linearly and using third order polynomals.

| Country | Age Category | Year-Max | Fitted - Max | Fitted 2013 | % Change | Rate Difference |
| --- | --- | --- | --- | --- | --- | --- |
|  | 65-74 y |  | (PMP) | (PMP) |  | (PMP) |
| High |  |  |  |  |  |  |
| US |  | 2006 | 1281.6 | 1128.3 | - 13.6 | - 153.3 |
| Greece |  | 2004 | 603.8 | 542.8 | - 11.2 | - 61 |
| Canada^$^ |  | 2004 | 648.6 | 588.9 | - 10.1 | - 59.7 |
| Belgium-Dutch |  | 2006 | 557.6 | 470.1 | - 18.6 | - 87.6 |
| Belgium-French |  | 2010 | 604.1 | 590 | - 2.4 | - 14.1 |
| Low |  |  |  |  |  |  |
| Austria |  | 2004 | 510.9 | 428.9 | - 19.1 | - 82 |
| Denmark |  | 2005 | 400.2 | 310.2 | - 29 | - 90 |
| Sweden |  | 2006 | 381.2 | 299.3 | - 27.4 | - 82 |
| The Netherlands |  | 2008 | 393.1 | 324.6 | - 21.1 | - 68.5 |
| Norway |  | 2007 | 355.9 | 299.4 | - 18.9 | - 56.5 |
| Finland |  | 2005 | 253.7 | 239.2 | - 6.1 | - 14.5 |
| Median(range) |  | 2006 (2004 to 2010) | 510.9 (253.7 to 1281.6) | 428.9 (239.2 to 1128.3) | -18.6 (-2.4 to -29) | -68.5  (-14.1 to -153.3) |
|  | ≥75 y |  |  |  |  |  |
| High |  |  |  |  |  |  |
| US |  | 2006 | 1545.8 | 1411.2 | - 9.5 | - 134.6 |
| Greece |  | 2013 | 911.3 | 911.3 | 0 | 0 |
| Canada^$^ |  | 2005 | 803.7 | 776.6 | - 3.5 | - 27.1 |
| Belgium-Dutch |  | 2009 | 938.8 | 891.4 | - 5.3 | - 47.4 |
| Belgium-French |  | 2008 | 866.2 | 802.9 | - 7.9 | - 63.2 |
| Low |  |  |  |  |  |  |
| Austria |  | 2005 | 528.6 | 478 | - 10.6 | - 50.6 |
| Denmark |  | 2007 | 470.4 | 448 | - 5 | - 22.3 |
| Sweden |  | 2007 | 426.8 | 375.2 | - 13.8 | - 51.6 |
| The Netherlands |  | 2010 | 478.9 | 447.3 | - 7.1 | - 31.6 |
| Norway |  | 2008 | 417.8 | 379.3 | - 10.2 | - 38.5 |
| Finland |  | 2004 | 246.6 | 193.7 | - 27.3 | - 52.9 |
| Median(range) |  | 2007 (2004 to 2013) | 528.6 (246.6 to 1545.8) | 478.0 (193.7 to 1411.2) | - 7.9 (0 to -27.3) | - 47.4 (0 to -134.6) |

^$^ Without Quebec
